# Supplementary material for: Urinary Markers of Oxidative Stress in Children with Autism Spectrum Disorder (ASD)
Source: Antioxidants (Basel). 2019 Jun 20;8(6):187. doi: 10.3390/antiox8060187 (PMC6616645; doi:10.3390/antiox8060187)
Supplement: Supplementary file 1 [file antioxidants-08-00187-s001.pdf]

R+ syntax for SVM's with linear, radial and polynomial kernel functions

```
# load library
library(caret)

# prepare formula
i <- 1
AvtBio_svm_formula[[i]] <- formula(Group ~ Octa.OH.diGvanozin.Kreat +
Izoprostan.Kreat + Di.Tirozin.Kreat + Hex.Lizin.Kreat)

# linear kernel
AvtBio_svm_Linear_control <- trainControl(method="optimism_boot",
number=1000, allowParallel = TRUE, classProbs=TRUE, sampling="smote")

AvtBio_svm_Linear_model <- list()
AvtBio_svm_Linear_results <- list()
AvtBio_svm_Linear_prediction <- list()
AvtBio_svm_Linear_prob <- list()
AvtBio_svm_Linear_confusion <- list()

AvtBio_svm_Linear_model[[i]] <- train(AvtBio_svm_formula[[i]],
data=AvtBio_data_svm[[i]], trControl=AvtBio_svm_Linear_control,
method="svmLinear", preProc = c("center", "scale"))

AvtBio_svm_Linear_results[[i]] <- AvtBio_svm_Linear_model[[i]]$results
AvtBio_svm_Linear_prediction[[i]] <-
predict(AvtBio_svm_Linear_model[[i]], AvtBio_data_svm[[i]])
AvtBio_svm_Linear_prob[[i]] <- predict(AvtBio_svm_Linear_model[[i]],
AvtBio_data_svm[[i]][,-1], type="prob")
AvtBio_svm_Linear_confusion[[i]] <-
confusionMatrix(AvtBio_svm_Linear_prediction[[i]],
AvtBio_data_svm[[i]]$Group)

# radial kernel
i <- 1
AvtBio_svm_Radial_control <- trainControl(method="optimism_boot",
number=1000, allowParallel = TRUE, classProbs=TRUE, sampling="smote")

AvtBio_svm_Radial_sigma <- list()
AvtBio_svm_Radial_tuning <- list()
AvtBio_svm_Radial_model <- list()
AvtBio_svm_Radial_results <- list()
AvtBio_svm_Radial_prediction <- list()
```

```
AvtBio_svm_Radial_prob <- list()
AvtBio_svm_Radial_confusion <- list()

AvtBio_svm_Radial_sigma[[i]] <- kernlab::sigest(AvtBio_svm_formula[[i]],
data=AvtBio_data_svm[[i]], frac = 0.5, na.action = na.omit, scaled =
TRUE)

AvtBio_svm_Radial_tuning[[i]] <- data.frame(expand.grid(C=c(0.01, 0.10,
0.20, 0.30, 0.40, 0.50, 0.60, 0.70, 0.80, 0.90, 1.00),
sigma=seq(from=AvtBio_svm_Radial_sigma[[i]][1],
to=AvtBio_svm_Radial_sigma[[i]][3], length.out=5)))

AvtBio_svm_Radial_model[[i]] <- train(AvtBio_svm_formula[[i]],
data=AvtBio_data_svm[[i]], trControl=AvtBio_svm_Radial_control,
method="svmRadial", preProc = c("center", "scale"),
tuneGrid=AvtBio_svm_Radial_tuning[[i]])

AvtBio_svm_Radial_results[[i]] <- AvtBio_svm_Radial_model[[i]]$results
AvtBio_svm_Radial_prediction[[i]] <-
predict(AvtBio_svm_Radial_model[[i]], AvtBio_data_svm[[i]])
AvtBio_svm_Radial_prob[[i]] <- predict(AvtBio_svm_Radial_model[[i]],
AvtBio_data_svm[[i]][,-1], type="prob")
AvtBio_svm_Radial_confusion[[i]] <-
confusionMatrix(AvtBio_svm_Radial_prediction[[i]],
AvtBio_data_svm[[i]]$Group)

# polynomial kernel
i <- 1
AvtBio_svm_Polynomial_control <- trainControl(method="optimism_boot",
number=1000, allowParallel = TRUE, classProbs=TRUE, sampling="smote")

AvtBio_svm_Polynomial_model <- list()
AvtBio_svm_Polynomial_results <- list()
AvtBio_svm_Polynomial_prediction <- list()
AvtBio_svm_Polynomial_prob <- list()
AvtBio_svm_Polynomial_confusion <- list()

AvtBio_svm_Polynomial_model[[i]] <- train(AvtBio_svm_formula[[i]],
data=AvtBio_data_svm[[i]], trControl=AvtBio_svm_Polynomial_control,
method="svmPoly", preProc = c("center", "scale"))

AvtBio_svm_Polynomial_results[[i]] <-
AvtBio_svm_Polynomial_model[[i]]$results
```

```
AvtBio_svm_Polynomial_prediction[[i]] <-
predict(AvtBio_svm_Polynomial_model[[i]], AvtBio_data_svm[[i]])
AvtBio_svm_Polynomial_prob[[i]] <-
predict(AvtBio_svm_Polynomial_model[[i]], AvtBio_data_svm[[i]][,-1],
type="prob")
AvtBio_svm_Polynomial_confusion[[i]] <-
confusionMatrix(AvtBio_svm_Polynomial_prediction[[i]],
AvtBio_data_svm[[i]]$Group)
```
